# Association between composite dietary antioxidant index and rheumatoid arthritis: results from NHANES 2003-2018

**DOI:** 10.7150/ijms.107332

**Published:** 2025-02-18

**Authors:** Qi Meng, Shankun Dong, Jianxun Ge, Chuanhong Qin, Guojiang Zhang, Chuanyun Fu, Shui Sun

**Affiliations:** 1 Department of Joint Surgery, Shandong Provincial Hospital, Shandong University, Jinan, Shandong 250012, China.; 2 Department of Joint Surgery, Shandong Provincial Hospital Affiliated to Shandong First Medical University, Jinan, Shandong 250021, China.; 3 Orthopaedic Research Laboratory, Medical Science and Technology Innovation Center, Shandong First Medical University & Shandong Academy of Medical Sciences, Jinan, Shandong 250117, China.; 4 Department of Stomatology, Shandong Provincial Hospital Affiliated to Shandong First Medical University, Jinan, Shandong 250021, China.; 5 School of Stomatology, Shandong First Medical University & Shandong Academy of Medical Sciences, Jinan, Shandong 250117, China.

**Keywords:** CDAI, NHANES, cross-sectional study, rheumatoid arthritis, oxidative stress.

## Abstract

**Background**: Increasing evidence has revealed oxidative stress as an essential risk factor in the development of rheumatoid arthritis (RA). Composite dietary antioxidant index (CDAI) is an important tool for assessing dietary antioxidant capacity. However, the association between CDAI and RA is still unclear.

**Method**: The data of 26501 participants from the NHANES database 2003-2018 cycle were collected to investigate the relationship between CDAI and RA risk. Logistic regression was used to examine the odds ratio (OR) of CDAI to RA risk. Restricted cubic spline (RCS) was utilized to test for potential nonlinear relationship. Stratified analysis and sensitivity analysis were also performed to strengthen the reliability of results.

**Results**: Multivariate logistic regression with full adjustment for covariates showed that the OR (95% confidence interval (CI)) for CDAI to RA was 0.974 (0.955, 0.993). A nonlinear negative correlation was identified by the RCS (p for nonlinearity=0.046). In both the subgroup and sensitivity analysis, this relationship was still present.

**Conclusion**: Our work suggests that higher dietary antioxidants intake is correlated with a lower RA incidence, thus providing some dietary recommendations for daily diets. Further clinical studies are acquired to better validate the current findings.

## Introduction

Rheumatoid arthritis (RA) is a major health problem worldwide 1. Globally, it is estimated that 17.6 million people are affected by RA, and prevalence of women is approximately 2.45 times higher than that of men 2. Currently, treatments for RA are categorized as non-surgical and surgical. Non-surgical treatment is suitable for the early stage, but it is unable to cure the disease 3, 4. When RA progresses to the late stage, appropriate surgical treatment is necessary, with total joint arthroplasty (TJA) being the last approach for severe patients 5, 6. However, due to the durability of the prosthesis and associated complications, TJA is not an optimal choice for middle-aged or active individuals 7, 8. Therefore, it is urgent to identify protective factors for RA to prevent the disease.

Oxidative stress (OS) is caused by excessive levels of reactive oxygen species (ROS) due to an imbalance between antioxidants and pro-oxidants 9-11. Previous studies have emphasized the important role of OS in the pathogenesis of RA, and weaker antioxidant capacity is associated with a higher risk of RA 12-14. Rheumatoid inflammation is accompanied by increased production of oxidants, which subsequently leads to oxidative damage to cartilage, tissue destruction and increased inflammation 15, 16. Conversely, antioxidant intake has been reported to help ameliorate the development of RA 17, 18. Composite dietary antioxidant index (CDAI) is a tool to evaluate an individual's dietary total antioxidants profile, which is based on the intake levels of multiple antioxidants (vitamins A, C, E, zinc, selenium and carotenoids) 19, 20. Previous researches have demonstrated that high CDAI is correlated with low mortality rates 21, 22. Moreover, CDAI exhibits a negative correlation with the development of several diseases, including hypertension 23, chronic kidney disease 24, diabetes 25, and osteoporosis 26. In addition, intaking antioxidants can also alleviate RA. Zhou *et al.* reported that dietary supplementation of vitamin A may have a potential preventive effect in RA 27. Regular vitamin E intake is effective for alleviating symptoms of RA 28. However, the relationship between CDAI and RA is currently unknown.

Our study used data from the National Health and Nutrition Examination Survey (NHANES) 2003-2018. We aimed to discover the potential association between CDAI and RA, with the goal to provide an appropriate dietary suggestion to prevent the disease.

## Method

### Data source and population

The NHANES is a series of sample surveys of representative health data from the general population of the United States conducted by the National Center for Health Statistics (NCHS). It employs a sophisticated, multistage stratified probability sampling design to collect demographics, dietary, examination, laboratory and questionnaire data and has been published biennially since 1999 29. The NHANES protocol was approved by the NCHS Research Ethics Review Board, and all participants provided informed consent.

**Figure 1:**
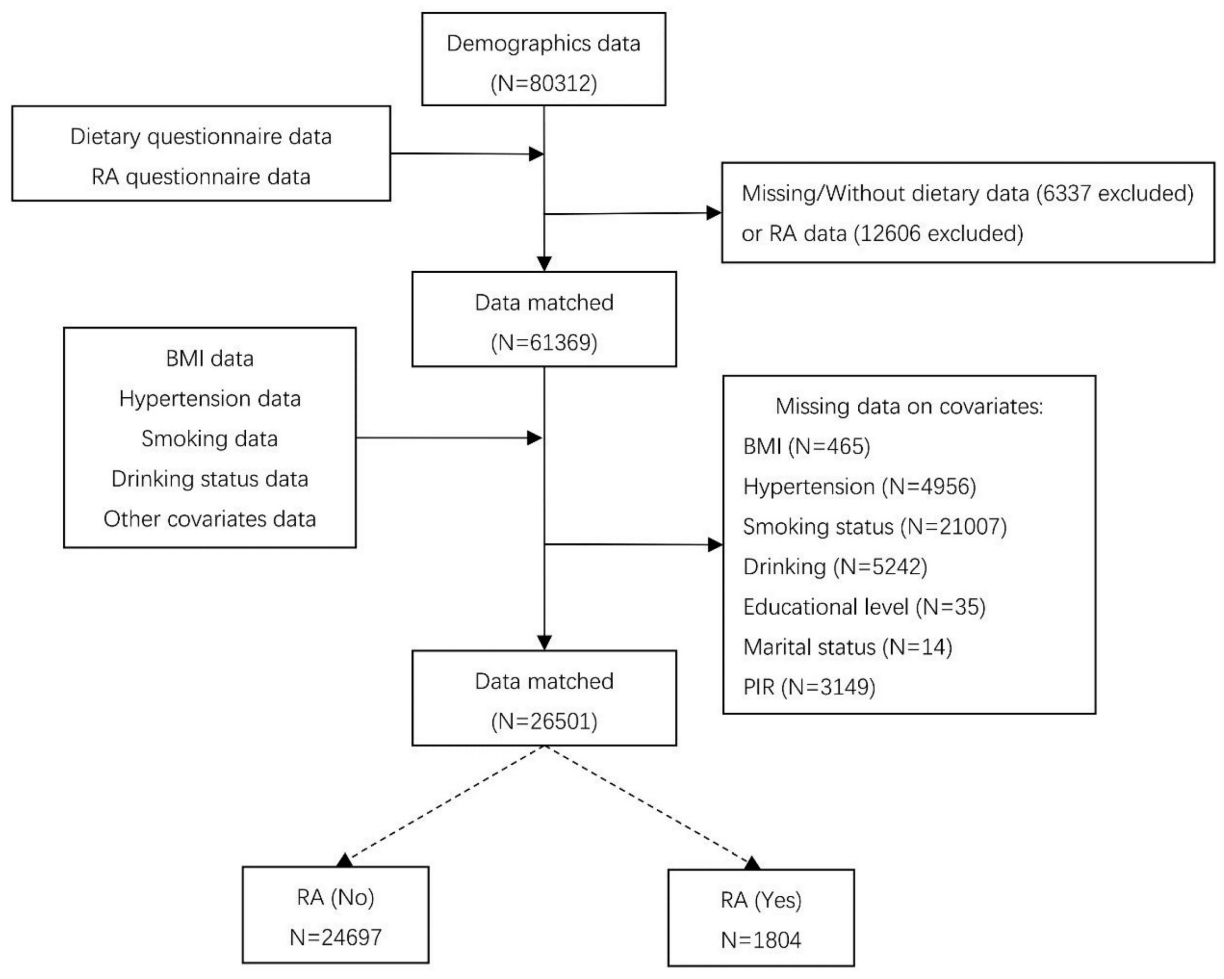
Flowchart of sample selection.

We used data from NHANES 2003-2018 cycle, which included detailed dietary and disease questionnaire information. In the 1999-2002 cycle, unfortunately, the carotenoid categories of the dietary questionnaire were not comprehensive compared to the 2003-2018 cycle and might cause analytic bias, so we discarded 1999-2002 cycle. Figure 1 illustrates the sample selection process. Totally, 80312 participants finished the survey during 2003-2018 cycle. We excluded individuals with missing dietary data (n=6337) or questionnaire information of RA (n=12606). In addition, participants with missing covariates were also excluded, including body measure index (BMI) (n=465), hypertension (n=4956), smoking status (n=21007), drinking status (n=5242), education level (n=35), marital status (n=14), and poverty-income ratio (PIR) (n=3149). Finally, 26501 subjects were included in our analysis.

### CDAI measurement

In NHANES, dietary intake was recorded for each participant by nonconsecutive 2-day 24-hour interviews. The first recording was performed at a mobile examination center, and the second was conducted online 3 to 10 days later. We extracted intake levels of 6 antioxidants (zinc, selenium, carotenoids, vitamin A, C, and E) based on these dietary recall data. CDAI is the sum of six standardized antioxidants intakes 19, 30, 31, as calculated below:



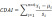



In this formula, x_i_ represents the daily antioxidants intake; µ_i_ represents the mean of x_i_; s_i_ represents the standard deviation (SD) of µ_i_.

### Outcome ascertainment

RA data was collected from personal interview data. Participants were asked if doctor told they had arthritis. If they answered "yes", the participant was then asked the type of arthritis and then categorized into RA, or other types of arthritis.

### Covariates

To evaluate the interference of potential confounding factors, this study included the following covariates: gender (Male, Female), age (<60, ≥60), race (Mexican American, Non-Hispanic White, Non-Hispanic Black, Other races) 32, educational level (Below High School, High School, Above High School) 33, marital status (married/living with partner, widowed/divorced/separated, never married) 34, PIR (<1.3, 1.3-3.5, ≥3.5) 30, total energy intake 35, hypertension status (Yes or No), BMI (<25.0, 25.0 to 30.0, ≥30.0) 36, alcohol consumption (Yes or No), and smoking status (Yes or No).

Gender, age, race, educational level, marital status and PIR information were obtained from demographics data. Total energy intake data was collected from dietary questionnaire. Hypertension was defined as self-reported hypertension or systolic blood pressure ≥140 mmHg or diastolic blood pressure ≥90 mmHg. Alcohol consumption was considered as “yes” if a participant had consumed alcohol, while BMI data was from body measurements profile. Smoking status was defined as “Yes”, if the participant had smoked.

### Statistical analyses

Continuous variables were expressed as mean (SD) and compared using independent samples t-tests. Categorical variables were described using frequency (percentage) and compared using the chi-square test or Fisher's exact test, as appropriate. CDAI was regarded as continuous variable or categorical variable converted by quartiles. Logistic regression analysis was performed to explore the relationship between CDAI and RA, in which three models were employed. Model I was a crude model with no confounding variables adjusted. Model II was adjusted for gender, age, race, educational level, marital status and PIR. Model III was further adjusted for total energy intake, hypertension, alcohol consumption, smoke status and BMI based on Model II. Then, Restricted cubic spline (RCS) was employed to analyze the nonlinear correlation between CDAI and RA. The correlation between CDAI and RA was further examined stratified by gender (male/female), age (<60/≥60 years), alcohol consumption (yes/no), hypertension (yes/no), BMI (<25, 25-30, ≥30), PIR (<1.3, 1.3-3.5, ≥3.5) and educational level (below high school, high school, above high school). Finally, we adopted a sensitivity analysis to evaluate the robustness of our results. RCSs were applied to investigate the relationship between each antioxidant and RA, respectively.

All analysis were conducted using SPSS (version 25.0) and R (version 4.2.3). P<0.05 was considered statistically significant.

## Results

### Population characteristics

26501 individuals were finally enrolled in the study, including 1804 participants with RA. Table 1 demonstrates the population baseline characteristics. Compared with non-RA individuals, RA patients tended to be female, over 60 years old, diagnosed with hypertension, have a higher BMI, and own less total energy. Significant differences existed in the distribution of race, education level, marital status, and PIR between RA and non-RA individuals. In addition, participants suffering from RA had significantly less intakes of all the antioxidants (vitamin A, vitamin C, vitamin E, zinc, selenium, and carotenoids) and lower CDAI level than non-RA participants.

### Relationship between CDAI and RA

Logistic regression models were established to explore the relationship between CDAI and RA. As mentioned in methods, three models were used in this study. As shown in Table 2, Continuous CDAI level was negatively associated with RA risk. In Model I, the odds ratio (OR) and 95% confidence interval (CI) were 0.930 (0.917,0.944; p<0.001), suggesting for per CDAI unit increase, the risk of RA reduced by 7.0%. This association was also significant in both Model II (OR: 0.970 (0.956, 0.985), p<0.001), and Model III (OR: 0.974 (0.955, 0.993), p=0.008) (Table 2). In addition, after dividing the CDAI levels into quartiles, the ORs of Q2 (Model I: 0.758 (0.669,0.858) p<0.001; Model II: 0.857 (0.753,0.976), p=0.020; Model III: 0.869 (0.758,0.996), p=0.044), Q3 (Model I: 0.630 (0.553,0.718), p<0.001; Model II: 0.811 (0.707,0.931), p=0.003; Model III: 0.834 (0.715,0.973), p=0.021), and Q4 (Model I: 0.491 (0.427,0.565), p<0.001; Model II: 0.735 (0.633,0.853), p<0.001; Model III: 0.774 (0.642,0.935), p=0.008) were also significantly lower in all three models compared to the Q1 (Table 2).

**Figure 2:**
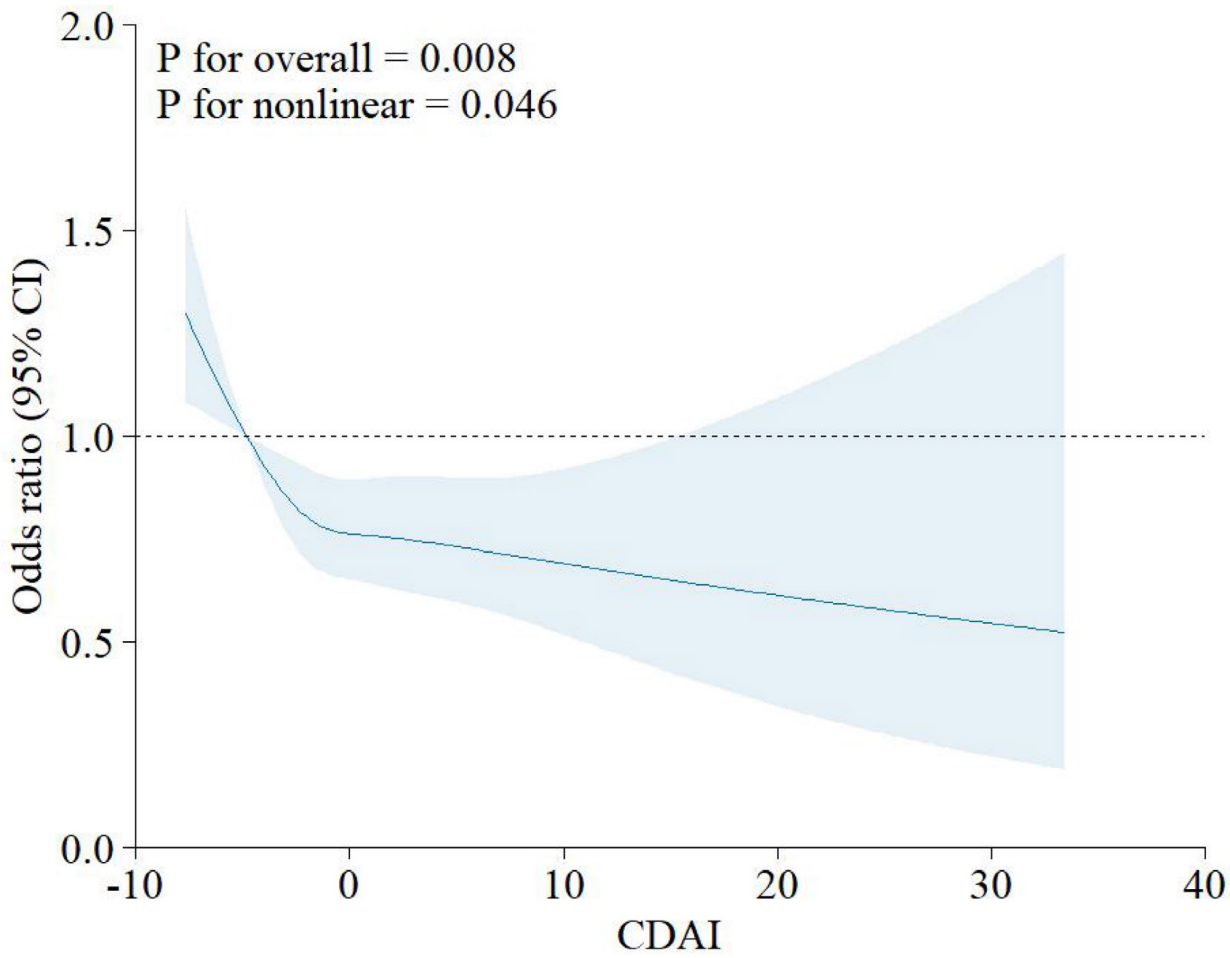
** RCS for the relationship between CDAI and RA.** Abbreviation: CDAI: composite dietary antioxidant index; CI: confidence interval.

In addition, RCS showed the relationship between CDAI and RA is non-linear (p for nonlinearity<0.046, Figure 2).

### Subgroup analysis

**Figure 3:**
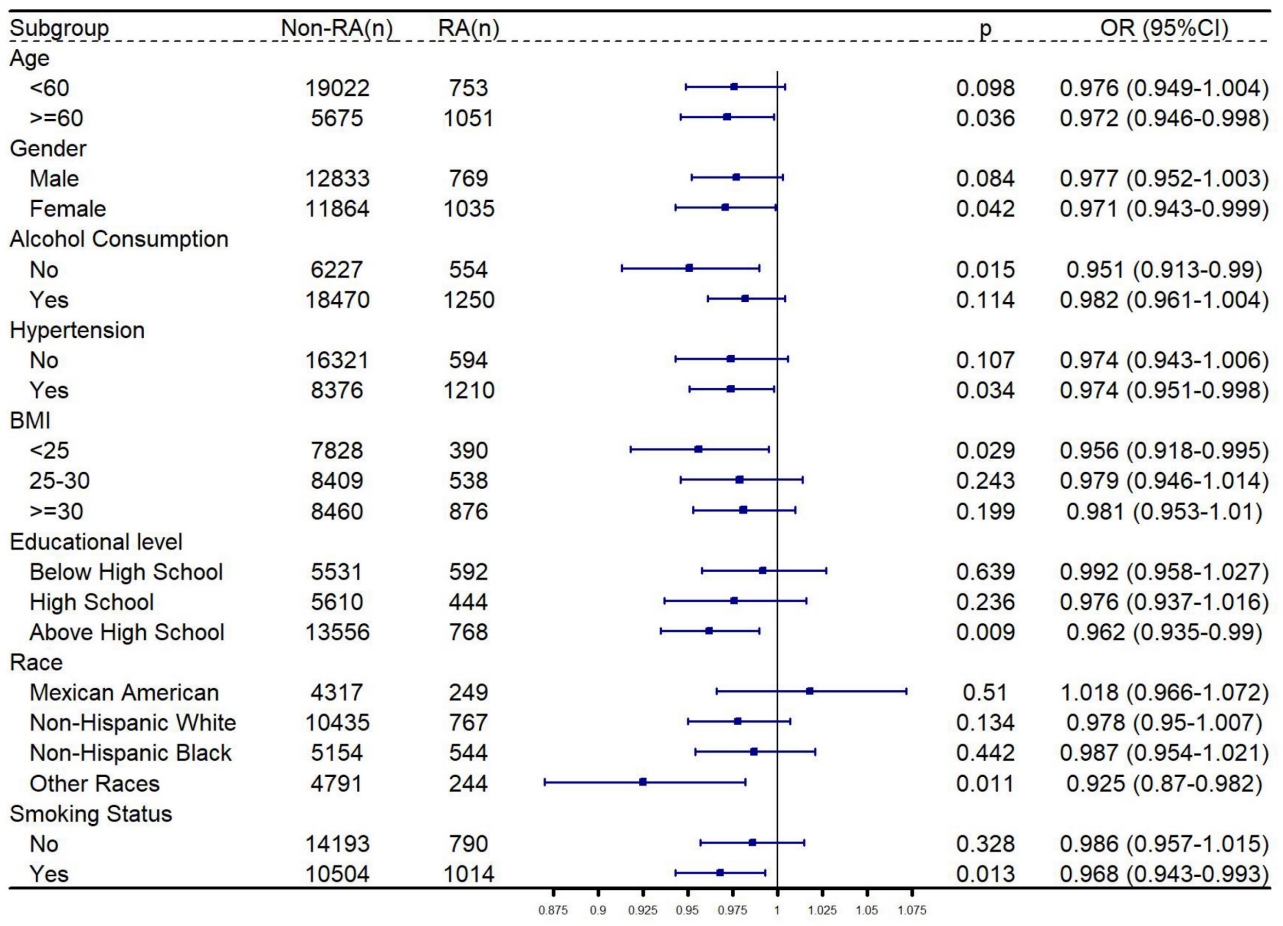
** Subgroup analysis for relationship between CDAI and RA.** Gender, age, race, educational level, marital status, PIR, total energy intake, hypertension, alcohol consumption, BMI and smoking status were adjusted, except the grouping criteria for each respective group (for example, “gender” was not adjusted in the gender-stratified group). Abbreviation: RA: rheumatoid arthritis; BMI: body measure index; PIR: poverty-income ratio; OR: odds ratio; CI: confidence interval.

The relationship between CDAI and RA was then explored in subgroup analysis. As shown in Figure 3, after adjusting the confounders, CDAI was significantly negatively related to RA among participants who were older than 60 years old, female, other races, had no history of alcohol consumption, no diagnosis of hypertension, and had a BMI < 25, highest level of education was high school, or had smoked.

### Sensitivity analysis

**Figure 4:**
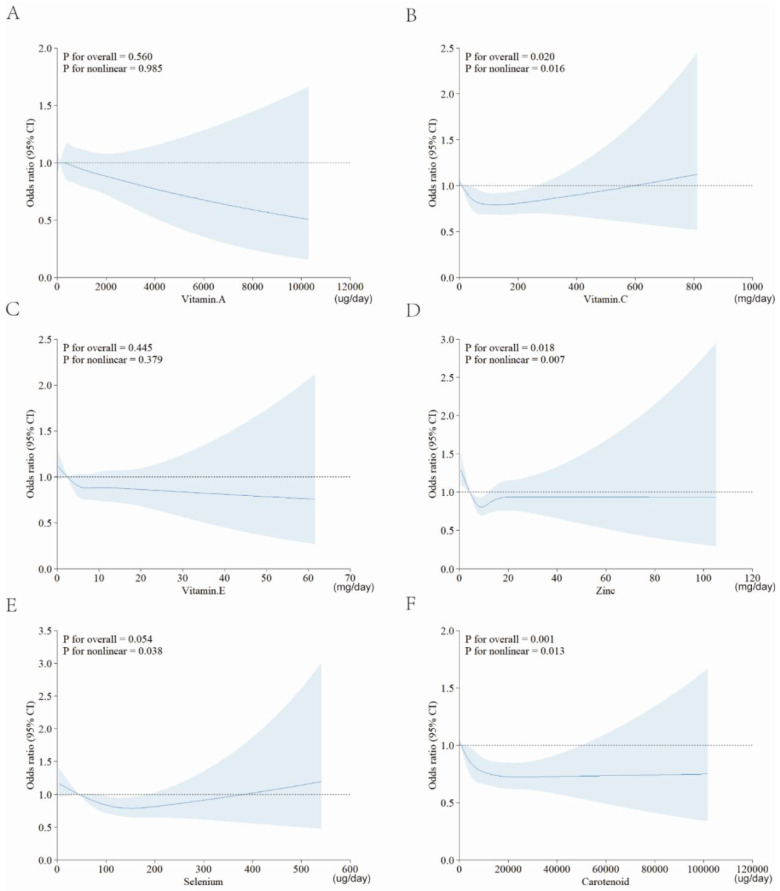
** RCS for the relationships between single antioxidant and RA.** A, vitamin A; B, vitamin C; C, vitamin E; D, zinc; E, selenium; F, carotenoid. Abbreviation: CDAI: composite dietary antioxidant index; CI: confidence interval.

To validate the robustness of the results, we conducted sensitivity analysis. As shown in Figure 4, we focused on the role of single antioxidant dietary supplement, and also applied RCSs to explore their association with RA. Except for vitamin A and vitamin E, there were still statistically significant nonlinear relationship between each antioxidant intake and RA risk.

## Discussion

In the present study, we examined relationship between CDAI and RA. After adjusting for possible confounding variables, we found that high CDAI level was correlated with lower risk of RA, suggesting that CDAI is a protective factor against RA. We then performed an RCS and found that this negative association was nonlinear. In addition, our subgroup analysis also demonstrated that in population who were older than 60 years old, female, other races, had no history of alcohol consumption, no diagnosis of hypertension, and had a BMI < 25, highest level of education was high school, or had smoked, CDAI was also significantly negatively associated with RA. In sensitivity analysis, dietary intake of vitamin C, zinc, selenium and carotenoid showed nonlinear correlations with RA. This is, to our knowledge, the first study to explore the relationship between CDAI (calculated using these six antioxidants) and RA using such a large sample.

OS occurs as a result of an imbalance between antioxidants and pro-oxidants and is rooted in excessive accumulation of ROS 9, 37. OS causes cellular damage in many tissues, which leads to the progression of many diseases 38-40, including orthopedic disorders 41, 42. Many studies have proved that OS can cause the bone homeostasis to tend to pathological states, and excessive ROS can lead to the apoptosis of osteocytes, osteoblasts and chondrocytes, and elevate osteoclasts formation and function 43-47. And OS also leads to inflammation via activating overproduction of some pro-inflammatory factors in the joints 48, 49. The cellular dysfunction and inflammatory environments will stimulate the development and progression of RA 48-50. As an external factor, diet is a feasible way to regulate the redox state and facilitate the prevention of various diseases 51-53. Some researches revealed the negative relationship between dietary antioxidants and RA, which is consistent with our study results. In active RA, the consumption of vitamin A, C, zinc and selenium is significantly and negatively correlated with some pro-inflammatory factors 54. Additionally, dietary supplement of vitamin A or vitamin E supplement might be a potential way to alleviate RA 27, 28. However, given the combination of food components, assessing the total dietary antioxidant intake may provide a more accurate and thorough understanding. Wang *et al.* calculated a dietary antioxidant index using intake levels of three vitamins and three metal ions and demonstrated a negative association with RA 55. In fact, there has been extensive studies on the relationship between CDAI and health or disease, and there are minor differences in the choice of the six antioxidants used for the calculation 55-61. In this study, we used intake levels of vitamins A, C, E, zinc, selenium, and carotenoids to calculate CDAI, in which carotenoids include α-carotene, β-carotene, cryptoxanthin, lycopene, lutein, and zeaxanthin. This type of calculation is also employed in a large number of studies and is used to explore correlations to multiple disorders 56, 57, 59, 61-63. High CDAI is correlated with low levels of cytokines involved in OS and inflammation, including TNF-α and IL-1β 64, 65. In young or middle-age adults and non-smokers, CDAI has the potential to reduce the risk of aging 66. Many studies have reported negative associations between CDAI and various diseases, including hypertension, diabetes, chronic kidney disease, hyperuricemia, depression, and heart failure 23-25, 31, 35, 67. Higher level of CDAI is also related to lower cardiovascular and mortality all-cause risk 22, 64, 68. In addition, several studies also reported that CDAI is positively related to bone mineral density, and prevents bone loss 69-72.

Multivariate logistic regression is powerful for studying the role of dietary factors played in disease development 73, 74. In our study, logistic regression showed that the OR of CDAI to RA was significantly lower than 1, suggesting CDAI as a potential protective factor. However, in the dose-effect curve of RCS, we find that this negative relationship is nonlinear. With rising CDAI, the risk of RA declined rapidly, then elevated mildly, followed by a slow decline. This indicates that keeping the CDAI at a proper level is profitable. In reality, it is not true that a higher intake of antioxidants is always better. Excessive supplementation can increase the risk of some disorders, including lung cancer and dyslipidemia 75, 76. In addition, excessive intake of some antioxidants sometimes increases the risk of arthritis. It is reported that excessive selenium intake is related to higher risk of osteoarthritis (OA), and that it may be safe to keep the daily intake below 100ug 77. In addition, people with a high zinc or selenium intake are also have an increased risk of developing OA, as indicated by Perri *et al.* 78. The CDAI was calculated by taking the intake of the six antioxidants, so we think that due to compositional interference, the risk of RA was not always decreasing as the CDAI increased. However, those with higher CDAI still showed fewer subjects with RA compared to the lowest quartile. Therefore, we believe that a low level of CDAI intake is not recommended, while moderate level of CDAI is preferable. Then, in order to increase the efficiency of data utilization and reveal the underlying truth, we conducted subgroup analysis. Without any confounders adjusted, a negative relationship between CDAI and RA could be found in every subgroup. After adjusting for all covariates in the study, this significant negative relationship existed among women, older than 60 years, without a history of alcohol consumption, without a diagnosis of hypertension, BMI < 25, those with the highest level of education at high school or had not smoked.

Our study has some advantages and shortcomings. The primary advantage is the large sample size, which makes our study more stable and convincing. Secondly, we explored the nonlinear relationship between CDAI and RA, providing evidence for rational dietary intake. However, several limitations should also be mentioned. Firstly, because of the cross-sectional layout, the study cannot clarify the causality between CDAI and RA. Secondly, although we tried to adjust for potential confounders, residual confounders might still exist. Additionally, we were unable to access disease severity in RA patients, which limited the exploration of the correlation of the CDAI with RA severity. Finally, the evaluation indicators were collected mainly from subjective questionnaires, and the individual dietary recall was conducted through two separate recall interviews, which may have been biased.

In conclusion, our cross-sectional study based on eight cycles (2003-2018) of NHANES database revealed that CDAI was negatively associated with RA. This study provides new avenues for exploring dietary interventions to reduce RA risk. Future randomized controlled trials or cohort studies are necessary to confirm this finding.

## Figures and Tables

**Table 1:** ** Population characteristics.** Data was presented as mean (SD) for continuous variables or frequencies (percentages) for categorical variables.

Characteristics		Overall	Non-RA	RA	P Value
N		26501	24697	1804	
Gender (%)	Male	13602 (51.3)	12833 (52.0)	769 (42.6)	<0.001^***^
	Female	12899 (48.7)	11864 (48.0)	1035 (57.4)	
Age (%)	<60	19775 (74.6)	19022 (77.0)	753 (41.7)	<0.001^***^
	≥60	6726 (25.4)	5675 (23.0)	1051 (58.3)	
Race (%)	Mexican American	4566 (17.2)	4317 (17.5)	249 (13.8)	<0.001^***^
	Non-Hispanic White	11202 (42.3)	10435 (42.3)	767 (42.5)	
	Non-Hispanic Black	5698 (21.5)	5154 (20.9)	544 (30.2)	
	Other	5035 (19.0)	4791 (19.4)	244 (13.5)	
Educational level (%)	<High School	6123 (23.1)	5531 (22.4)	592 (32.8)	<0.001^***^
	High School	6054 (22.8)	5610 (22.7)	444 (24.6)	
	>High School	14324 (54.1)	13556 (54.9)	768 (42.6)	
Marital status (%)	Married/Living with partner	16150 (60.9)	15165 (61.4)	985 (54.6)	<0.001^***^
	Widowed/Divorced/Separated	4956 (18.7)	4287 (17.4)	669 (37.1)	
	Never married	5395 (20.4)	5245 (21.2)	150 (8.3)	
PIR (%)	<1.3	8085 (30.5)	7364 (29.8)	721 (40.0)	<0.001^***^
	1.3-3.5	10023 (37.8)	9341 (37.8)	682 (37.8)	
	≥3.5	8393 (31.7)	7992 (32.4)	401 (22.2)	
Vitamin A(µg/day) (mean (SD))		612.37 (588.23)	614.96 (589.31)	576.97 (572.22)	0.008^**^
Vitamin C (mg/day) (mean (SD))		86.58 (82.79)	87.20 (82.90)	78.09 (80.86)	<0.001^***^
Vitamin E (mg/day) (mean (SD))		7.89 (5.41)	7.96 (5.46)	6.98 (4.58)	<0.001^***^
Zn (mg/day) (mean (SD))		11.47 (7.03)	11.56 (7.08)	10.28 (6.10)	<0.001^***^
Se (µg/day) (mean (SD))		113.64 (55.25)	114.60 (55.31)	100.51 (52.71)	<0.001^***^
Carotenoid (µg/day) (mean (SD))		9466.24 (10293.96)	9568.64 (10377.93)	8064.27 (8952.00)	<0.001^***^
CDAI (mean (SD))		0.13 (4.04)	0.19 (4.06)	-0.76 (3.67)	<0.001^***^
Energy(kcal/day) (median [IQR])		2106.03 (883.65)	2123.21 (886.42)	1870.82 (809.15)	<0.001^***^
Hypertension (%)	No	16915 (63.8)	16321 (66.1)	594 (32.9)	<0.001^***^
	Yes	9586 (36.2)	8376 (33.9)	1210 (67.1)	
Alcohol Consumption (%)	No	6781 (25.6)	6227 (25.2)	554 (30.7)	<0.001^***^
	Yes	19720 (74.4)	18470 (74.8)	1250 (69.3)	
BMI (%)	<25	8218 (31.0)	7828 (31.7)	390 (21.6)	<0.001^***^
	25-30	8947 (33.8)	8409 (34.0)	538 (29.8)	
	≥30	9336 (35.2)	8460 (34.3)	876 (48.6)	
Smoke (%)	No	14983 (56.5)	14193 (57.5)	790 (43.8)	<0.001^***^
	Yes	11518 (43.5)	10504 (42.5)	1014 (56.2)	

*p<0.05, **p<0.01, ***p<0.001. Abbreviation: RA: rheumatoid arthritis; PIR: poverty-income ratio; SD: standard deviation; CDAI: composite dietary antioxidant index; BMI: body measure index.

**Table 2:** ** Association between CDAI and RA.** Model I: no covariates were adjusted. Model II: gender, age, race, educational level, marital status and PIR were adjusted. Model III: gender, age, race, educational level, marital status, PIR, BMI, total energy intake, hypertension, alcohol consumption, and smoking status were adjusted.

CDAI	Model I		Model II		Model III	
	OR (95%CI)	P value	OR (95%CI)	P Value	OR (95%CI)	P Value
Continuous	0.930 (0.917,0.944)	<0.001^***^	0.970 (0.956, 0.985)	<0.001^***^	0.974 (0.955, 0.993)	0.008^**^
Quartile 1	Reference					
Quartile 2	0.758 (0.669,0.858)	<0.001^***^	0.857 (0.753,0.976)	0.020^*^	0.869 (0.758,0.996)	0.044^*^
Quartile 3	0.630 (0.553,0.718)	<0.001^***^	0.811 (0.707,0.931)	0.003^**^	0.834 (0.715,0.973)	0.021^*^
Quartile 4	0.491 (0.427,0.565)	<0.001^***^	0.735 (0.633,0.853)	<0.001^***^	0.774 (0.642,0.935)	0.008^**^

*p<0.05, **p<0.01, ***p<0.001. Abbreviation: CDAI: composite dietary antioxidant index; OR: odds ratio; CI: confidence interval.

## Abbreviations

RA: rheumatoid arthritis
CDAI: Composite Dietary Antioxidant Index
OR: Odds Ratio
RCS: Restricted Cubic Spline
CI: Confidence Interval
TJA: Total Joint Arthroplasty
NHANES: National Health and Nutrition Examination Survey
NCHS: National Center for Health Statistics
BMI: Body Measure Index
PIR: Poverty-Income Ratio
SD: Standard Deviation
OS: Oxidative Stress
ROS: Reactive Oxygen Species
OA: Osteoarthritis
